# Time-dependent affective disturbances in abstinent patients with methylphenidate use disorder

**DOI:** 10.1186/s12888-022-04187-5

**Published:** 2022-08-22

**Authors:** Jie Xu, Yi Zhang, Nan Wang, Pei Sun, Fuqiang Mao, Ti-Fei Yuan

**Affiliations:** 1 Department of Psychiatry and Psychology, Tianjin Medical University, Tianjin, China; 2 Beijing Gaoxin Hospital, Beijing, China; 3 Department of Psychology, Tsinghua University, Beijing, China; 4 Shanghai Key Laboratory of Psychotic Disorders, Shanghai Mental Health Center, Shanghai Jiaotong University School of Medicine, Shanghai, China; 5 Co-Innovation Center of Neuroregeneration, Nantong University, Nantong, Jiangsu China; 6 Shanghai Key Laboratory of Anesthesiology and Brain Functional Modulation, Translational Research Institute of Brain and Brain-Like Intelligence, Shanghai Fourth People’s Hospital Affiliated to Tongji University School of Medicine, Shanghai, China

**Keywords:** Methylphenidate, Ritalin, Addiction, Depression, Anxiety, Adolescents

## Abstract

**Background:**

Methylphenidate (MPH), also called Ritalin, is used to treat attention-deficit hyperactivity disorder (ADHD) patients. With occasional reports of subjects suffering from Methylphenidate use disorder (MPHUD), few studies analyzed the neuropsychological changes in this population.

**Purpose:**

This study aims to evaluate the clinical outcomes of individuals with MPHUD.

**Methods:**

We retrospectively analyzed 61 MPH patients (aged 16–27 years) admitted to the Beijing Gaoxin Hospital drug rehabilitation program from Jan 2017 to Mar 2019. The drug use history and drug abuse motivation scale were collected at admission. Clinicians rated the Hamilton Depression Rating Scale, Hamilton Anxiety Rating Scale, and DSM-5 Stimulant use disorder criteria each week during the 4 weeks rehabilitation program. Correlation analyses were conducted between drug use history and affective disturbances.

**Results:**

The results showed that the adolescent period is the peak for MPH exposure, and 1/3 of patients got their first exposure to MPH from their parents. MPH abstinence accompanies severe anxiety and depression symptoms, significantly alleviating after four weeks of treatment.

**Conclusions:**

MPHUD is associated with substantial affective disturbances, which warrants a more considerable sample investigation.

## Introduction

Methylphenidate (MPH), also called Ritalin, is a central nervous system (CNS) stimulant which increases dopamine concentration by blocking the reuptake transporter [1]. Clinically, MPH has been approved for attention-deficit hyperactivity disorder (ADHD), attention deficit disorder (ADD), and narcolepsy [2]. In addition, MPH may be considered a replacement therapy for methamphetamine dependence patients [3, 4].

MPH is considered a weak stimulant at clinical doses and does not elicit sufficient dopamine release for reinforcing effects [1]. On the other hand, the potential of intravenous Methylphenidate use disorder (MPHUD) has been suggested [5, 6], which is further evidenced by [^18^F]DOPA PET imaging on pharmacokinetics in the striatum [7]. According to the global report of 2021, 5.7% of respondents are accompanied by MPHUD [8]. In China, methylphenidate has been listed as a first-class psychotropic drug by the National Medical Products Administration (NMPA) since 2007. However, there has been a rapid increase in the non-medical use of prescribed medicine in China (estimated prevalence of 6% in South China), including methylphenidate [9]. In the United States, there has been a growing tendency of non-medical use of MPH among high school students in the past 30 years, the prevalence of non-medical use of Ritalin is around 0.5% in 2021 [10]. A Meta-analysis reported that the proportion of non-medical use of Ritalin is approximately 16.4% in Iran [11]. In most cases, MPHUD is reported as injecting or sniffing, with very few reports on oral administration[6].

Anxiety and depression are the most common withdrawal symptoms of substance use disorder [12]. Previous studies indicated that the depressive symptoms of patients with methamphetamine use disorder displayed an acute phase of 7–10 days followed by a subacute phase of up to 3 weeks [13, 14]. In the acute phase, symptoms' severity decreased linearly from the initial level on the first day of methamphetamine abstinence [13, 14]. In the subacute phase, symptoms were relatively mild and stable for around two weeks [13, 14]. In MPH, merely have studies investigated the withdrawal symptoms of MPHUD. Although previous studies have claimed the potential side effects of long-term MPH use [5, 6], such as psychotic symptoms, impaired neurocognitive functioning, agitation, and mood alterations[6], whether the depressive symptoms of MPH use display a similar pattern of methamphetamine remains unclear. Moreover, more significant affective symptoms during abstinence are related to higher suicide attempts, worse treatment adherence, and a higher relapse rate [14–16]. Thus, understanding the affective symptoms during abstinence of MPHUD may help to guide the targeted treatment and prevent further relapse.

Occasional reports of subjects suffered from MPHUD, and few studies analyzed the neuropsychological changes in this population. To evaluate the clinical outcomes of MPHUD individuals, we retrospectively analyzed the mood status and other clinical characteristics of 61 subjects with MPHUD through oral administration.

## Method

### Clinical data

We retrospectively analyzed 61 MPHUD patients (aged 16–27 years) admitted to the Beijing Gaoxin Hospital drug rehabilitation program from Jan 2017 to Mar 2019. Clinicians obtained patients' medication history through self-report or from their parents. There was. All patients had a long history of MPH oral administration at high doses, exhibited withdrawal symptoms after cessation, and had no concurrent use of other medications. Inclusion criteria required that participants met the Structured Clinical Interview for the Diagnostic and Statistical Manual of Mental Disorders (DSM-5) diagnosis of stimulant use disorder (moderate or severe) as assessed by board-certified psychiatrists. Exclusion criteria were (1) diagnosed history of bipolar disorder, major depressive disorder, anxiety disorder, schizophrenia, schizoaffective disorder, other psychotic disorders, current psychotic symptoms, or other neurologic diseases; (2) currently had a physical disorder or neurologic disease; (3) met the diagnosis of other major psychiatric diseases as assessed by board-certified psychiatrists; (4) comorbid with other substance use disorder, such as heroin, methamphetamine, and cocaine. No subject had attention-deficit/hyperactivity disorder (ADHD) or any other psychiatric disease. The study has been approved by the Ethics Committee of Beijing Gaoxin Hospital (ethics committee approval number: GXYYLLWYH201910086). All participants provided written informed consent.

### Neuropsychological evaluations

Upon admission to the hospital, the drug use history and drug abuse motivation scale [17] were collected by board-certified psychiatrists. Clinicians rated the Hamilton Depression Rating Scale, Hamilton Anxiety Rating Scale, and DSM-5 Stimulant use disorder criteria each week during the 4 weeks rehabilitation program.

For DSM-5 Stimulant use disorder criteria, the total score of 0–2 is considered as being normal, the presence of 2–3 symptoms considered mild, 4–5 moderate, and over 6 indicated severe symptoms.

The drug abuse motivation scale is a self-rating scale used to evaluate the motivation for drug use. The scales include 32 items on a 5-point scale (1 represents "totally disagree," 5 means "completely agree"). The scale has six dimensions (Cronbach α: 0.88) [17]: social pressure, drug use values, environmental factors, physical symptoms, negative emotion, and high sensation-seeking. A higher score indicates a higher level of drug abuse motivation. Cronbach's alpha for the drug abuse motivation scale was 0.626 in the present study.

Hamilton Depression Rating Scales (HAMD)-17 scale [18] is widely used in assessing depressive severity. The scales include 17 items on a 5-point scale (0 represents "none", 4 represents "extremely severe") [19, 20]. A trained rater screens the scale by conversation and observation. A total score of 0–7 is considered normal, 8–16 suggest mild depression, 17–23 moderate depression, and over 24 indicates severe depressive symptoms. HAMD-17 contains five factors: (1) anxiety/somatic, (2) weight, (3) cognitive dysfunction, (4) stuck, (5) sleep difficulty. Cronbach's alpha for HAMD was 0.652 in the present study.

Hamilton Anxiety Rating Scales (HARS) [21] is widely used in assessing the anxiety severity [20, 22]. The scales include 14 items on a 5-point scale (0 represents "none," and 4 illustrates "extremely severe"). A trained rater screens the scale by conversation and observation. A total score of 0–6 indicates a normal condition, 7–13 mild anxiety, 14–20 moderate anxiety, and larger than 21 suggests a severe anxiety condition. Cronbach's alpha for HARS was 0.710 in the present study.

### Statistical analysis

All data analysis was performed using R Studio 1.0.153.0 (RStudio, Inc) and SPSS v. 21(IBM Corp., NY, USA). Pearson and Spearman's correlation was used to compare the relationship between demographic information and the clinical outcomes of participants. Kernel Density Curve Estimation explored the distribution of participants' demographic data, such as age, Body Mass Index (BMI), and age of first drug use. Pearson correlation was used to compare the correlation between baseline and changes in anxiety and depression scores. The Linear Regression model was employed to investigate the association between drug use history and depression as well as anxiety scores of participants. The linear regression model also explored the impact factor of daily dosage. The overall statistical significance threshold was set as two-tailed, *p* < 0.05.

## Result

### Demographics of participants

The demographic information is presented in Table 1. Most patients were males (51 out of 61). The mean age was 19.607 years old (SD = 1.891). More than half of the participants had a high school education (42 participants), and 14 had a junior high school education. Two third of the participants live in the city (*n* = 41), and one-third live in the rural areas (*n* = 20). 18 out of 61 patients were introduced to MPH firstly by parents, 19 by friends, and 24 by themselves. The most common reason for beginning to use MPH was to improve study performance.

**Table 1:** Demographic and clinical characteristics of participants

	**Mean**	**Standard deviation**		**N**
Gender	61: 10 females; 51 males			
Marriage	Single: 55; married: 2; divorced: 4			
Education level	Primary:4; junior high:14; high:32; undergraduate:10; unclear:1			
Occupation	Jobless: 22; self-employed: 2; farmer: 12; other: 24			
Sites	Rural area:20; city: 41			
Provence	Northeast: 51; Shandong:1; Shanxi:1; Tianjing:6; Wuhan:2			
Sources of first use	Parents given:18; friends given: 19; themselves:24			
Age (year)	19.607		1.8910	61
Addiction years	4.410		2.8070	61
Age of first use	16.803		2.1257	61
Daily dosage	5.984		2.6863	61
BMI	23.288		2.2823	61
DSM-5	9.426		0.8388	61

**Fig. 1:**
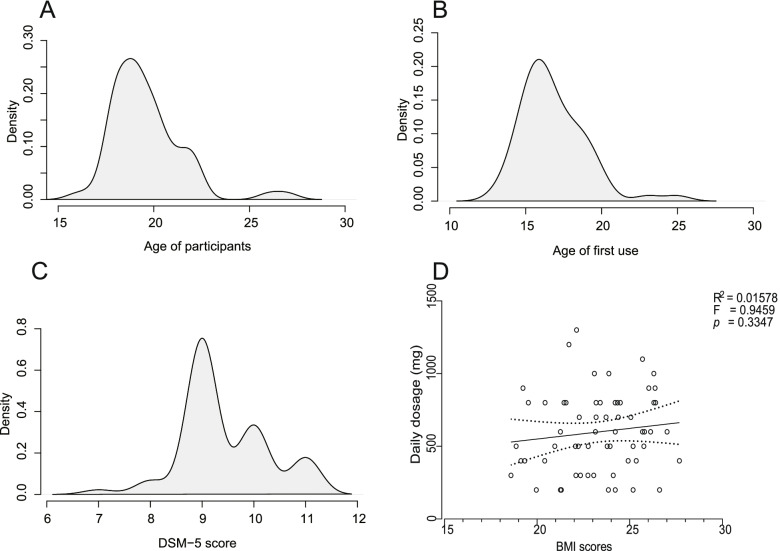
Density plot for participants' age (**A**) age of first use (**B**) DSM-5 score (**C**) and linear regression between BMI score and daily dosage (**D**)

The patients exhibited severe symptoms of substance use disorder (DSM-5) score of more than six (M = 9.426, SD = 0.8388) (Fig. 1A), which are mainly 18–20 years old (Fig. 1B). The first use of MPH mainly occurs at 14–18 years old (M = 16.803, SD = 2.1257) (Fig. 1C), which is at the high school admission stage. In present population, we did not detect a positive correlation between BMI and the intake dosage (gram) (*r* = 0.126, *p* = 0.3347) (Fig. 1D).

### Alterations of mood states during abstinence

**Fig. 2:**
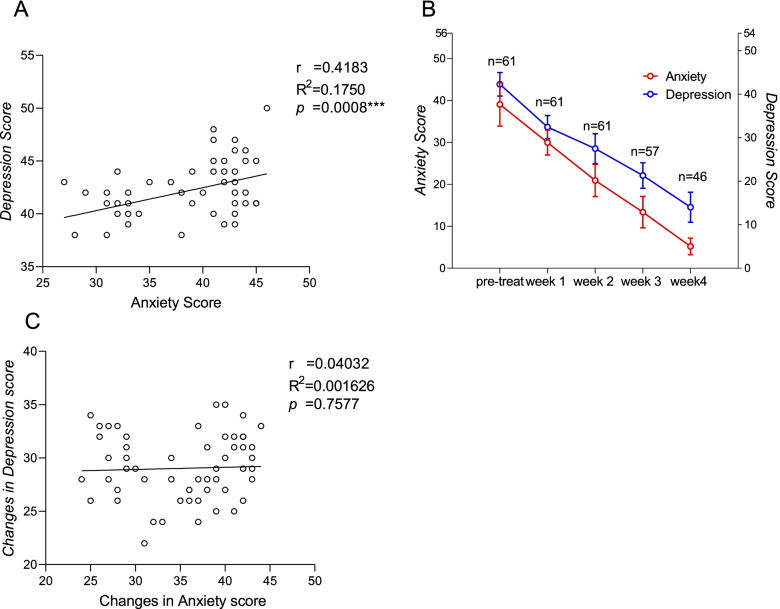
Correlation between depression and anxiety score in Methylphenidate use dependences (**A**) the tendency of anxiety score and depression score during the treatment, the left y axe means anxiety score, and the right y axe means depression score (**B**) the correlation between changes in depression score and anxiety score during the treatment (**C**)

The patients exhibited a high level of anxiety and depression upon admission, with a significant correlation to each other (*R* = 0.418, *p* < 0.001) (Fig. 2A). Four participants were discharged after two weeks of treatment, 11 were discharged after three weeks of treatment, and the remaining 46 were discharged after receiving four weeks' treatment. During the abstinence period, anxiety and depression scores steadily decreased (Fig. 2B). The repeated measure ANOVA was conducted for anxiety and depression changes, and significant main effects were observed (Anxiety: F (1,45) = 1684.265, *p* < 0.001; Depression: F (1,45) = 2444.350, *p* < 0.001). Notably, the changes in anxiety and depression scores were not correlated (R = 0.04032, *p* = 0.758) (Fig. 2C).

With linear regression analyses, daily dosage did not show any association with depression score and anxiety score (F (1,59) = 1.719, *p* = 0.195 and F (1,59) = 0.0007677, *p* = 0.978, respectively). Addiction years showed a slightly negative association in both depression scores and anxiety scores but did not significant (F = 1.871, *p* = 0.177, and F = 2.276, *p* = 0.137, respectively).

### Impact factors for daily dosage

**Fig. 3:**
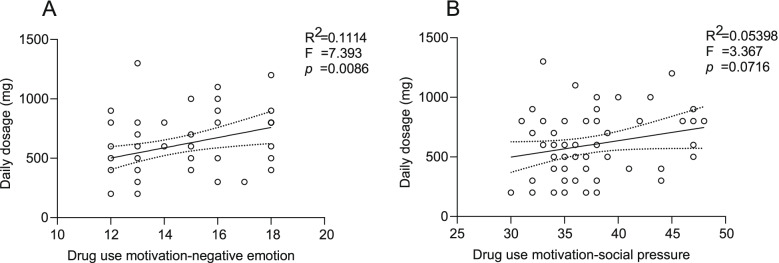
Linear regression analysis for daily dosage and Drug abuse motivation-negative emotion factor(**A**) Daily dosage and drug abuse motivation-social pressure factor(**B**)

We found that the negative emotion factor in the drug abuse motivation scale depicted significant positive regression with daily dosage (*F* (1,59) = 7.393, R^2^ = 0.111, *p* = 0.0086) (Fig. 3A). The social pressure factor in the drug abuse motivation scale showed marginally positive regression with daily dosage (F (1,59) = 3.367, *p* = 0.0716) (Fig. 3B).

## Discussion

The present study reported oral MPHUD with severe mood disturbances during abstinence. As the abstinence prolongs, the depression and anxiety symptoms show alleviation. Time-dependent affective disturbances have been reported in the abstinence period for different substance abusers, including methamphetamine, alcohol, nicotine, and heroin [13, 14]. The abstinence accompanied by mood disturbances could implicate targeted management of these MPHUD patients.

Disruption of mesolimbic dopamine transmission, neurotrophic factor decreases, and altered opioid receptor signaling were implicated in the altered affective processing in the abstinence period, including other psychostimulant abusers (e.g., methamphetamine) [23–25]. Indeed, withdrawal from MPH exposure increases midbrain neural activity and alters the stress sensitivity brain regions [26]. Other clinical cases reported depression, fatigue, loss of appetite, and even movement disorders (e.g., dystonia) in MPH abstinence [27, 28]. The current study showed a similar pattern of depression and anxiety reduction in the first month of abstinence compared with previous studies that examined methamphetamine use disorder [13, 14]. However, the depressive and anxiety symptoms still showed linear reduction and did not remain stable at 2–3 weeks. These differences may be due to different mechanisms of psychostimulant effects of the two drugs [29]. Currently, no study directly compares the neural mechanisms differences between MPH and methamphetamine. However, in the animal study, a recreational dose of MPH, but not methamphetamine, can reduce the anxiety-like behavior [30]. Interestingly, the present population showed correlated depression and anxiety scores at baseline, while the changes in depression scores did not correlate with changes in anxiety. Future studies should elucidate further the detailed symptom arsenal and neural mechanisms underlying MPH abstinence.

The results demonstrated a positive correlation between negative emotion, social pressure, and the amount of daily dosage in these subjects. A previous study reported that MPH had been regarded as a cognitive enhancement (CE) [31]. Most subjects likely began to take MPH to increase study/work capacity [32, 33], aiming to relieve school competition [31] and productivity-related demands [34–36]. All these results suggested that academic stress might facilitate the formation of MPHUD [31]. Besides, the lack of relevant knowledge leads to a misunderstanding of Ritalin in parental populations. Therefore, they hold the wrong belief that Ritalin can facilitate the academic performance of their children with no harm [31]. It further promotes the abuse of Ritalin.

The dose-dependence effect on anxiety and depression was not observed in this study. Previous studies indicated that the dosage and route of drug administration significantly impacted withdrawal scores [37, 38]. Injectors and high dosage participants showed higher withdrawal severity [38]. Smokers with higher cigarettes consumptions have shorter withdrawal latency [39]. The lack of the dose-dependent effect may be due to the insufficient measure of withdrawal symptoms. We only measured the anxiety and depression scores. Therefore, it may weaken the potential dose-dependent influence.

The study is limited in several aspects. First, the cross-sectional research covered four weeks of the in-hospital period. A longer follow-up is preferred for outcome observation and potentially dissecting the effect of affection state on relapse probabilities. Besides, current findings are based on screening tools, more information may be obtained through clinical interviews. Secondly, it will be helpful to perform neuroimaging studies on these patients in future studies to elucidate the potential structural and functional changes in the brain network underlying MPHUD. Last but not least, comparing these patients with other psychostimulants (e.g., methamphetamine) dependents in terms of clinical symptom severity and other neuropsychological behaviors will be interesting.

## Summary

The present study retrospectively analyzed MPHUD patients and observed severe mood disturbances among patients with MPHUD during abstinence. These negative emotional statuses are essential predictors for abuse severity and could alleviate after four-week treatment, implicating targeted management of these MPHUD patients.

## Data Availability

The datasets generated and/or analyzed during the current study are not publicly available due to limitations of ethical approval involving the patient data and anonymity but are available from the corresponding author on reasonable request.
